# Efficacy of pegylated interferon α-2a and α-2b in patients with genotype 1 chronic hepatitis C: a meta-analysis

**DOI:** 10.1186/1471-2334-12-357

**Published:** 2012-12-18

**Authors:** Nicola Coppola, Mariantonietta Pisaturo, Gilda Tonziello, Caterina Sagnelli, Evangelista Sagnelli, Italo F Angelillo

**Affiliations:** 1 Department of Public Medicine, Section of Infectious Diseases, Second University of Naples, Naples, Italy; 2 Department of Experimental Medicine, Section of Hygiene, Second University of Naples, Naples, Italy; 3 Division of Infectious Diseases, Azienda Ospedaliera Sant’Anna e San Sebastiano di Caserta, Caserta, Italy; 4 Department of Clinical and Experimental Medicine and Surgery, “F. Magrassi e A. Lanzara” Second University of Naples, Naples, Italy

**Keywords:** Antiviral therapy in HCV patients, Meta-analysis, Response to anti-HCV therapy, Tolerability of anti-HCV therapy

## Abstract

**Background:**

Two formulations of Pegylated interferon (Peg-IFN) are on the market for treatment of chronic hepatitis C virus (HCV) infection. The purpose of this meta-analysis was to assess the efficacy of Peg-IFN α-2a versus Peg-IFN α-2b in combination with ribavirin in anti-human immunodeficiency virus (HIV)-negative patients with genotype 1 chronic HCV infection.

**Methods:**

The following criteria were to be met for inclusion in the meta-analysis: (a) original data from randomized and non-randomized clinical trials; (b) study on the efficacy of conventional doses of Peg-IFN α-2a (180 μg/week) versus Peg-IFN α-2b (1.5 μg/kg of body weight/week), both in combination with ribavirin, in antiviral therapy-naïve HCV-genotype 1 subjects; (c) at least one of these primary outcomes: Rapid Virological Response (RVR); Early Complete Virological Response (EVR); End of Treatment Response (ETR); Sustained Virological Response (SVR); (d) odds ratio estimates of relative risk (RR) and associated 95% confidence intervals (CIs) or at least data enabling them to be computed; (e) English language; and (f) published as a full paper up to December 2011.

**Results:**

Seven published studies met the inclusion criteria, allowing a meta-analysis on 3,026 patients. Peg-IFN α-2a and Peg-IFN α-2b showed similar rate of RVR (RR = 1.05; 95% CI = 0.87-1.27, *p* = 0.62) and SVR (RR = 1.08; 95% CI = 0.99-1.18, *p* = 0.098). Peg-IFN α-2a more frequently than Peg-IFN α-2b achieved EVR (RR = 1.11; 95% CI = 1.02-1.21, *p* = 0.013) and ETR (RR = 1.22; 95% CI = 1.14-1.31, *p* < 0.0001).

**Conclusion:**

The standard schedules of Peg-IFN α-2a and Peg-IFN α-2b, both in combination with ribavirin, can be used indifferently for patients with chronic HCV genotype 1 who are anti- to eliminate HIV-negative and antiviral treatment-naïve.

## Background

It is well known that chronic hepatitis C virus (HCV) infection is responsible for acute hepatitis progressing to chronicity in about 70% of cases [1]. Half of the patients with chronic HCV infection require antiviral treatment with a combined administration of Pegylated interferon (Peg-IFN) α and ribavirin [2,3]. For patients with HCV genotype 2 or 3, the treatment is based on a 24-week administration of Peg-IFN given once a week in combination with a daily oral dose of 800 mg of ribavirin, whereas for those with HCV genotype 1 or 4, a 48-week combined treatment with a daily dose of ribavirin between 13–15 mg/kg is prescribed [4-6]. These treatment schedules allow a sustained virological response in nearly 80% of patients with HCV genotype 2 or 3 and in 40-45% of those with HCV genotype 1 [2].

Two formulations of Peg-IFN are on the market, Peg-IFN α-2a, given at a weekly standard dose of 180 μg, and Peg-IFN α-2b, given at a dose of 1.5 μg per kg of body weight. The identification of the best Peg-IFN for chronic HCV is of minor relevance for patients with genotype 2 or 3 since most of them achieve Sustained Virological Response (SVR) with either Peg-IFN formulation [4-6], whereas patients with HCV genotype 1 show a much lower rate of SVR [4-6] and, consequently, are at a higher risk of progression to cirrhosis or hepatocellular carcinoma. A difference in the SVR rates between the two Peg-IFN formulations is of substantial clinical impact for genotype 1 patients.

Previous studies have compared the effect of the two formulations, but the results are not conclusive. The purpose of this meta-analysis was to provide a systematic review of all randomized and non-randomized trials in which the efficacy of Peg-IFN α-2a had been compared with that of Peg-IFN α-2b, both in combination with ribavirin, in the treatment of patients with genotype 1 chronic HCV. This will provide reliable guidance for clinical practice and future research, particularly in the light of the therapy combinations with directly acting antivirals soon to be released on the market.

## Methods

The Quality of Reporting of Meta-analyses guidelines have been followed throughout the design, implementation, analysis, and reporting of this meta-analysis [7].

### Search strategy

A comprehensive systematic literature search of computerized bibliographic databases including MEDLINE, EMBASE, LILACS, and the Cochrane Library, from January 2000 to December 2011, was carried out on the efficacy of Peg-IFN α-2a versus Peg-IFN α-2b in combination with ribavirin in patients with genotype 1 chronic HCV. The search was conducted using both medical subject heading (MeSH) terminology and more general search terms. Search terms included, but were not limited to: Pegylated interferon α-2a, Pegylated interferon α-2b, HCV infection, HCV-related chronic hepatitis, treatment of HCV-related chronic hepatitis, Peg-IFNα-2a versus Peg-IFNα-2b in the treatment of HCV-related chronic hepatitis. Additionally, reference lists of the selected papers and the review articles on this topic were manually scanned to identify any other pertinent studies.

### Inclusion and exclusion criteria

The following criteria were necessary for inclusion in the meta-analysis. The studies had to: (a) present original data from randomized or non-randomized trials; (b) investigate the efficacy of conventional doses of Peg-IFN α-2a (180 μg/week) versus Peg-IFN α-2b (1.5 μg/kg of body weight/week), both in combination with ribavirin, in antiviral therapy-naïve HCV-genotype 1 subjects; (c) report at least one of the primary outcomes clearly defined as Rapid Virological Response (RVR), HCV RNA-negative after 4 weeks of treatment; Early Complete Virological Response (EVR), HCV RNA-negative after 12 weeks of treatment; End of Treatment Response (ETR), HCV RNA-negative on completion of treatment; and SVR, undetectable HCV RNA 6 months after therapy completion; (d) report data allowing to calculate the odds ratio estimates of relative risk (RR) for the effect on different outcomes of therapy with Peg-IFN α-2a (180 μg/week) versus Peg-IFN α-2b (1.5 μg/kg/week), both in combination with ribavirin; (e) be written in English; and (f) be published as a full paper up to December 2011. Studies were excluded if they were observational or they included patients who had undergone liver transplantation or were anti-human immunodeficiency virus (HIV)-positive.

Two investigators (NC and MP) independently screened title, abstract, and key words of each reference identified and filled out an inclusion/exclusion form for all papers. Full copies of included papers were then retrieved and independently reviewed for eligibility by the two investigators; contrasting opinions were analyzed by both investigators and unanimous consensus was reached after discussion.

### Data extraction

Standard information was extracted independently and in duplicate from each selected study by two investigators (NC and MP). The data sought included years the study was performed, baseline patient characteristics, treatments received, duration of follow-up, types and numbers of evaluations during the follow-up, risk ratios (RRs) and standard errors (SEs) of these estimates. If the latter were not available, they were calculated. Any disagreement between the investigators was resolved as mentioned above.

### Quality assessment

Two investigators (NC and IFA) independently assessed the quality of the included trials using the Jadad et al. [8] and the Chalmers et al. [9] methods. The Jadad et al. scale analyses the criteria related to the randomization methods (0 to 2 points), double-blinding (0 to 2 points), and withdrawals (1 point). A numerical score between 0 and 5 was assigned as a measure of study design and reporting quality, with 0 being the weakest and 5 designating the strongest. The Chalmers et al. method gives a handicap to the score of each item according to whether it has been completely (full score), partially (half score) or not at all (no score) addressed. If an item in the protocol was not applicable, the number of possible points was reduced, thus adapting the scoring system to different situations. The score for each paper was calculated as the ratio of the total points assigned divided by the total number of points considered applicable to that study, yielding a range from 0 to the perfect score of 1. Any disagreement in quality assessment between the two investigators was resolved as mentioned above.

### Statistical analysis

In the primary analysis, all included trials were considered. The pooled-effects estimates were used to combine the values from the single studies and were expressed as RR and 95% confidence interval (CI). RR and CI were obtained using the Mantel-Haenszel fixed-effects model [10] if the studies were homogeneous, and the random-effects models according to the method described by DerSimonian & Laird [11] in cases with heterogeneity. Random-effects models incorporate variation both within and between studies and typically provide wider CIs when heterogeneity is present. The secondary analysis was carried out in order to identify any influence of the design and of the quality of the trials on the findings obtained. Therefore, a sensitivity analysis was carried out to identify any influence of the non-randomized evidence, by grouping only the randomized trials, and of the quality of the studies, by classifying reports according to a Jadad et al. score greater than or equal to the median.

In order to assess statistical heterogeneity, the data were reanalyzed using both random- and fixed-effects models. Cochrane’s Q statistics was used to assess whether differences in the results were comparable with chance alone. Since this test has low power when only few studies or studies with a low sample size are included in a meta-analysis, a *p*-value < 0.10 was regarded to indicate significant heterogeneity. The I^2^ measure was also used [12]. The I^2^ measure is the percentage of total variation across studies that is attributable to heterogeneity rather than chance, where values approaching zero (0%) indicated no observed heterogeneity and larger values increasing heterogeneity. The random-effects model results were applied in case of I^2^ equal or higher than 50%. Heterogeneity was also assessed through visual inspection of L’Abbé plots.

To assess the potential for publication bias, funnel plots were constructed for each outcome in which the log RRs were plotted against their SEs. The Begg rank correlation test was used to examine the association between the effects estimates and their variances [13], and the Egger linear regression test, which regresses z statistics on the reciprocal of the SE for each study, was used to detect publication bias [14].

All statistical analyses were done with Stata [15].

All the procedures used in the study wherein accordance with the International Guidelines with the standards on human experimentation of the Ethics committee of “Azienda Ospedaliera Universitaria of the Second University of Naples” and with the Helsinki Declaration of 1975 revised in 1983.

## Results

### Study characteristics

**Figure 1:**
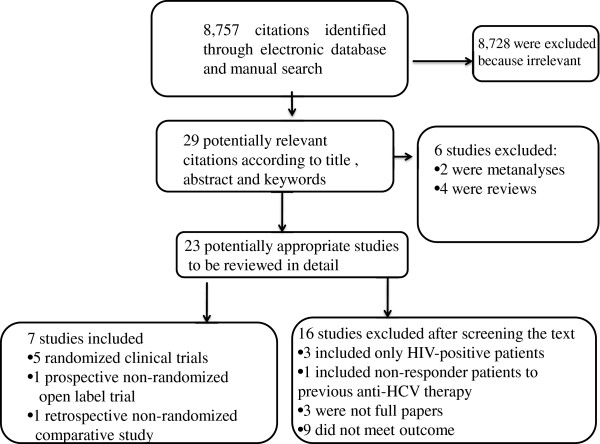
Flow chart of the published studies evaluated for inclusion in the meta-analysis.

A total of 8,757 citations were identified through electronic database and manual search using the above-reported keywords. Of these 8,757 citations, 29 were considered potentially relevant but only 7 [16-22] met the inclusion criteria and were included in the meta-analysis (Figure 1).

Tables 1 and 2 show the characteristics of the studies included in the meta-analysis. Five of them were prospective randomized trials [16,17,19,20,22], one prospective non-randomized [18], and one retrospective non-randomized [21]. The studies, published between 2006 and 2010, included from 37 to 2,054 patients. In all studies, the ribavirin dose was weight-based ranging from 800 to 1,400 mg and was reduced or discontinued, respectively, when the hemoglobin level was <10 g/dL or below 8.5 mg/day. The studies had different approaches for the reduction of ribavirin: one trial reduced the dose to 600 mg/day [16], one trial by 200 mg/day (in patients receiving 800–1,200 mg/die ribavirin) or by 400 mg/day (in patients receiving 1,400 mg/die) in the Peg-IFN α-2b arm, and to 600 mg/die in the Peg-IFN α-2a arm [19]; in two trials it was reduced by 200 mg/die for both regimens [20,22]. All studies reported the data as Intention-To-Treat analysis, but one study provided data by Per Protocol analysis [16]. One study was included although the data on the virological outcome had been presented for genotypes 1 and 4 [20].

**Table 1:** Characteristics of the clinical trials included in the meta-analysis

**First Author [Reference No.]**	**Country**	**Enrolment period**	Study*	**Analysis**	**No. of patients**	**Age, years, mean**	**Gender,% male**
					**Peg-IFN**	**Peg-IFN**	**Peg-IFN**
					**α-2a/α-2b**	**α-2a/α-2b**	**α-2a/α-2b**
Yenice [16]	Turkey	No information	RCT, unblinded	Per protocol	37/37	48.2 and 50.9^	35.1/27
						50.8 and 50.85§	
Di Bisceglie [17]	USA	No information	RCT, unblinded	Intention-to-treat	189/191	46.9/48.4	64/71
Escudero [18]	Spain	May 2001 to December 2006	Controlled	Intention-to-treat	59/58	45.8/45.1	66.1/60.3
McHutchison [19]	USA	March 2004 to June 2006	RCT^**#**^	Intention-to-treat	1,035/1,019	47.6/47.5	59.2/60.2
Ascione [20]	Italy	March 2004 to December 2006	RCT, unblinded	Intention-to-treat	93/93	51.3/48.9°	50.6/58.8°
Lee [21]	Korea	May 2004 to February 2009	Controlled	Intention-to-treat	21/16	53.4/53.6°	64.6/55.3°
Rumi [22]	Italy	September 2003 to June 2007	RCT, unblinded	Intention-to-treat	91/87	51.6/52.8°	60.4/54.8

*RCT randomized controlled trial; **#** Random double-blinded with regard to the dose of Peg-IFN alfa-2b; ^ Male; § Female; ° All genotypes.

**Table 2:** Outcomes in the studies included in the meta-analysis

**First Author [Reference No.]**	**Outcomes, n° (%) of patients in Peg-IFN α-2a/α-2b group**						
	**RVR**	**EVR**	**ETR**	**SVR**	**Discontinuation of therapy for adverse events in patients with genotype 1 all genotypes**		**Peg-IFN reduction for adverse events in patients with all genotypes**
Yenice [16] #			28(75.7)/27(73)	18(48.6)/13(35.1)	3 (7.5)/3 (7.5)	3 (7.5)/3 (7.5)	
Di Bisceglie [17] §	14(7.4)/22(11.5)	74(39.1)/84(43.9)					
Escudero [18] †	15(25.4)/10(17.2)	33(55.9)/29(50)	12(20.3)/7(12.1)	30(50.8)/27(46.6)		12(13.1)/10(10.8)	8(8.7)/7(7.6)
McHutchison [19] $	123(11.9)/116(11.4)	466(45)/407(39.9)	667(64.4)/542(53.2)	423(40.9)/406(39.8)	135(13)/129(12.6)	135(13)/129(12.6)	264(25.5)/254(24.9)
Ascione [20] §				51(54.8)/37(39.8)	3 (3.2)/13 (14)	4(2.5)/22(13.7)	
Lee [21] ^		16(76.2)/12(75)	16(76.2)/13(81.3)	8(38)/10(62.5)		8(10.1)/5(10.6)	
Rumi [22] ç	34(37)/26(30)	60(66)/40(46)	59(65)/38(44)	44(48)/28(32)		16(7.5)/17(7.7)	22(10.3)/14(6.3)

Dosage of ribavirin

# 40**–**64 kg of weight: 800 mg/day; 65–85 kg: 1,000 mg/day; >85 kg: 1,200 mg/day.

§ <75 kg of weight: 1,000 mg/day; ≥75 kg: 1,200 mg/day.

† ≤65 kg of weight: 800 mg/day; 65–75 kg: 1,000 mg/day; >75 kg: 1,200 mg/day.

$ In the Peg-IFN α-2b arm: 40–65 kg of weight: 800 mg/day; >65-85 kg: 1,000 mg/day; >85-105 kg: 1,200 mg/day; >105-125 kg: 1,400 mg/day. In the Peg-IFN α-2a arm: <75 kg: 1,000 mg/day; ≥75 kg: 1,200 mg/day.

^ In the Peg-IFN α-2a arm: <75 kg of weight: 1,000 mg/day; ≥75 kg: 1,200 mg/day. In the Peg-IFN α-2b arm: <65 kg: 800 mg/day; 65–85 kg: 1,000 mg; >85 kg: 1,200 mg.

ç <75 kg of weight: 1,000 mg/day; ≥75 kg: 1,200 mg/day.

### Assessment of quality

Table 3 summarizes the methodological quality assessment of the studies included in the meta-analysis. The mean overall quality scores of the individual studies using the Chalmers et al. scale ranged from 0.2 to 0.6 (mean = 0.43), for the Protocol from 0.11 to 0.58 (mean = 0.36) and for the Data Analysis and Presentation from 0.23 to 0.67 (mean = 0.53). All trials received full credit for the description of inclusion and rejection criteria for patient selection, analysis of results of randomization, and number of patients who withdrew and the reasons why. Most trials tested the validity of randomization, reported and discussed side effects of treatment, indicated the start and stop dates, estimated variance and/or confidence limits of the endpoints, and used regression/correlation analysis. In none of the studies, the observers were masked to the treatment and results, none presented test statistics and *p*-value, or discussed beta error. According to the quality criteria set forth by Jadad et al., none of the studies had scores of 5 or 4, three trials scored 3, two scored 1, and two trials scored 0.

**Table 3:** Distribution of studies by quality-scoring values according to the Chalmers et al. and Jadad et al. methods

	**Chalmers**																									**Jadad score**			
**First Author [Reference No.]**	1	2	3	4	5	6	7	8	9	10	11	**Protocol**	12	13	14	15	16	17	18	19	20	21	22	**Data analysis**	**Overall**	1	2	3	**Overall**
Yenice [16]	1	0	1	0	0	0	0	0	0	1	0	0.17	0	1	0	0	0	0	0	1	0	0	0	0.23	0.2	1	0	0	1
Di Bisceglie [17]	1	0	1	0	0	0	0	1	1	1	n.a.	0.35	0	1	0	0	1	0	0	1	0	1	0	0.44	0.39	1	0	0	1
Escudero [18]	1	0	1	0	0	0	0	0	0	0	0	0.11	1	1	0	0	1	1	0	1	0	1	0	0.61	0.31	0	0	0	0
McHutchison [19]	1	1	1	1	0	0	0	1	1	1	0	0.58	1	1	0	0	1	1	0	1	0	1	0	0.61	0.6	2	0	1	3
Ascione [20]	1	1	1	1	0	0	0	1	1	1	0	0.54	1	1	0	n.a.	1	1	0	1	0	1	0	0.67	0.59	2	0	1	3
Lee [21]	1	0	1	0	0	0	1	0	0	1	0	0.15	1	1	0	0	0	1	0	1	0	1	0	0.54	0.37	0	0	0	0
Rumi [22]	1	1	1	1	0	0	0	1	1	1	0	0.54	1	1	0	n.a.	1	1	0	1	0	1	0	0.67	0.59	2	0	1	3

For the Chalmers et al. method: 0 is “Non adequate”, 1 is “Adequate” for the following items: 1 Selection description; 2 Number and reasons for eligible patients not included in the study; 3 Regimen definition; 4 Blinding of Randomization; 5 Blinding of Patients to therapy; 6 Blinding of Physicians/observers to therapy; 7 Blinding of Physicians/observers to ongoing results; 8 Regimen definition; 9 Statistical estimate of sample size; 10 Testing randomization; 11 Testing compliance; 12 Dates of study; 13 Results of prerandomization; 14 Both test statistics and *P* value given; 15 Post beta estimate; 16 Confidence intervals given; 17 Regression/correlation; 18 Statistical analysis; 19 Number and reasons for patients withdrawn after randomization; 20 Withdrawals handled in several ways; 21 Side effects discussion; 22 Subgroups retrospective analysis. For the Jadad et al. the points are assigned for the following items: 1 Randomization; 2 Double-blinding; 3 Withdrawals and drop-out.

*n*.*a*. not applicable.

### Effects of interventions

Table 4 shows the overall treatment effect of Peg-IFN α-2a versus Peg-IFN α-2b according to the different outcomes of interest. The rate of RVR, assessed in 4 studies with 2,729 patients, was similar in the Peg-IFN α-2a and Peg-IFN α-2b groups. The EVR and ETR were both evaluated in five studies with a number of patients respectively of 2,766 and 2,460; the Peg-IFN α-2a group achieved more frequently than the Peg-IFN α-2b EVR (RR = 1.11; 95% CI = 1.02-1.21, *p* = 0.013) and ETR (RR = 1.22; 95% CI = 1.14-1.31, *p* < 0.0001). Six studies assessed the SVR (2,646 patients) and no significant difference was observed between the Peg-IFN α-2a and Peg-IFN α-2b groups.

**Table 4:** Summary of meta-analysis results in the achievement of the virological outcome by Pegylated interferon α-2a and α-2b plus ribavirin in patients with genotype 1 chronic hepatitis C

**Outcomes**	**N° of studies**	**N° of patients**	**N° and (%) of events**	**RR (efficacy)**	**95% CI (efficacy)**	***p***	**Heterogeneity test**
		**α-2a/α-2b**	**α-2a/α-2b**				**(Q;*p*;I**^**2**^**,%)**
Rapid Virological Response	4 [17-19,22]	1,374/1,355	186(13.5)/174(12.8)	1.05	0.87–1.27	0.62	3.8;0.28;21.1
Early Virological Response	5[17-19,21,22]	1,395/1,371	649(46.5)/572(38.4)	1.12	1.03–1.22	0.011	7.58;0.14;42.8
End of Treatment Response	5[16,18,19,21,22]	1,243/1,217	782(62.9)/627(51.5)	1.22	1.14–1.31	<0.0001	7.6;0.11;47.3
	5^*^[16,18,19,21,22]	921/879	544(59)/403(45.8)	1.29	1.18–1.41	<0.0001	8.37;0.08;52.1
Sustained Virological Response	6[16,18-22]	1,336/1,310	574(43)/521(39.8)	1.08	0.99–1.18	0.098	9.97;0.08;49.8
	6^**^[16,18-22]	1,058/732	473(44.7)/306(41.8)	1.08	0.97–1.20	0.19	10.9;0.053;54.2
	6^*^[16,18-22]	1,014/972	406(40)/346(35.5)	1.13	1.01–1.26	0.04	8.59;0.13;41.8

* In McHutchison’s study [19] only patients without ribavirin reduction were included.

** In McHutchison’s study [19] only patients with adequate ribavirin dosage (≥13 mg/kg/die) were included.

The analysis by subgroups was performed since the trial conducted by McHutchison et al. [19] reported the data on ETR and SVR according to the dosage and the reduction of ribavirin. The analysis including only the group of patients who received an adequate daily dose of ribavirin (≥13 mg/kg/day) showed no significant difference between Peg-IFN α-2a and Peg-IFN α-2b in the achievement of SVR. Moreover, in the analysis including only the patients who did not receive a reduction in the dosage of ribavirin during treatment, Peg-IFN α-2a had a significantly better effect than Peg-IFN α-2b on ETR (RR = 1.29; 95% CI = 1.18-1.41, *p* < 0.0001) and SVR (RR = 1.13; 95% CI = 1.01-1.26, *p* = 0.04).

The sensitivity analyses, performed by excluding the trials deemed to be of poor methodological quality and by excluding the non-randomized trials, revealed that the overall findings regarding all outcomes were not affected and no significant differences were observed in the pooled-effect estimates, thus confirming the robustness of the results of the meta-analysis. No homogeneity test showed statistically significant heterogeneity.

Some of the studies described the adverse events (AE) leading to the discontinuation of antiviral treatment or a reduction in the Peg-IFN dose. Three studies including 2,320 patients reported the discontinuation of treatment, but no significant difference was observed between the Peg-IFN α-2a and Peg-IFN α-2b groups (RR = 0.96; 95% CI = 0.77-1.19, *p* = 0.7; test for heterogeneity Q = 5.61, *p* = 0.06; I^2^ = 64.4%) regarding discontinuation. It was not possible to evaluate the rate of AE leading to a reduction in the Peg-IFN dose, since only one trial reported these data [19]. The rate of AE leading to discontinuation of antiviral therapy or to a reduction in Peg-IFN dose was also evaluated in the included studies considering patients with genotype 1 and non-1. The AE leading to the discontinuation of Peg-IFN were reported in 6 studies (3,194 patients) and those leading to Peg-IFN reduction in 3 (2,668 patients); no significant differences were found between Peg-IFN α-2a and Peg-IFN α-2b regarding the occurrence of AE leading to discontinuation (RR = 0.93; 95% CI = 0.77-1.13, *p* = 0.48; test for heterogeneity Q = 10.65, *p* = 0.06; I^2^ = 53.1%) or to reduction in the Peg-IFN dosage (RR = 1.06; 95% CI = 0.92-1.22, *p* = 0.45; test for heterogeneity Q = 1.92, *p* = 0.38; I^2^ = 0%).

**Figure 2:**
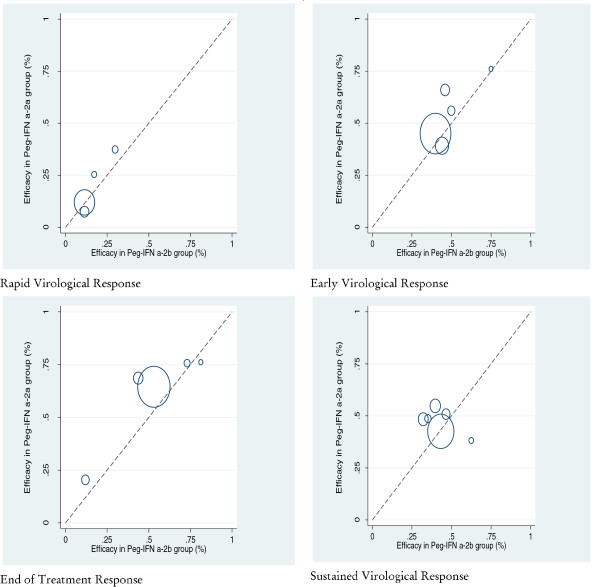
L’Abbé plot for efficacy rate in Peg-IFN α-2a and Peg-IFN α-2b groups on the different outcomes of interest.

Visual analysis of L’Abbé plots for the efficacy rate in the Peg-IFN α-2a and Peg-IFN α-2b groups of different outcomes gave no evidence of heterogeneity in the studies included in the meta-analysis (Figure 2).

**Figure 3:**
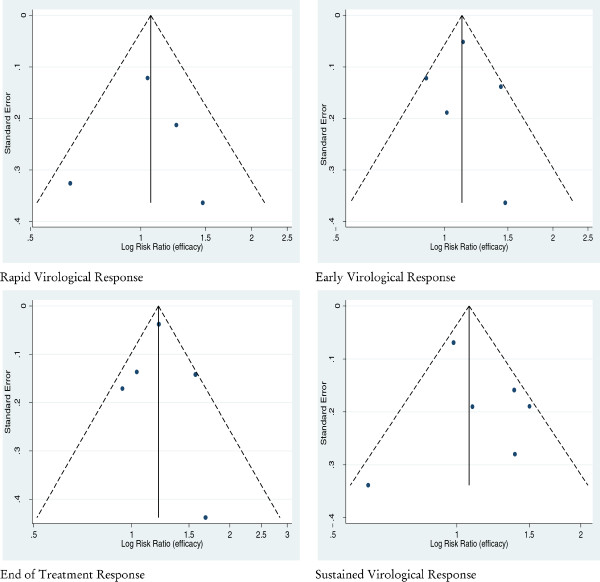
Funnel plot of the risk ratios vs. the reciprocal of their standard errors of trials evaluating the efficacy of Peg-IFN α-2a versus Peg-IFN α-2b on the different outcomes of interest.

A funnel plot of the effect size versus standard error from each study was generated to evaluate the presence of a potential publication bias with regard to the meta-analysis performed. Visual analysis of the funnel plots at all stages of the review and the rank correlation or regression testing gave no evidence of publication bias in the studies included in the meta-analysis. Neither Egger’s test (*p* > 0.20) nor Begg’s test (*p* > 0.20) was significant and gave no evidence for publication bias (Figure 3).

## Discussion and conclusion

This meta-analysis summarized the results of studies comparing Peg-IFN α-2a with Peg-IFN α-2b administered weekly subcutaneously in combination with a daily dose of ribavirin for therapy-naïve anti-HIV-negative patients with genotype 1 chronic HCV. The data show that there is no difference between the two treatments in the achievement of RVR and SVR; the higher rates of EVR and ETR in the patients treated with Peg-IFN α-2a are clearly of lesser clinical impact. The datum on SVR may be considered the most important result from this meta-analysis. In fact, it is well known that SVR is the most reliable indicator of HCV eradication; a follow-up study on more than 1,300 patients with SVR achieved with Peg-IFN α, alone or in combination with ribavirin, found that the recurrence of HCV RNA was rarely recorded after a mean of 3.9 years [23].

As already stated, some of the previously conducted studies showed no difference between the two treatments in the rate of SVR in therapy-naïve patients with HCV genotype 1 [16,18,19]; in other studies, SVR was observed more frequently with Peg-IFN α-2a [20,22]; finally, in one trial the patients treated with Peg-IFN α-2b achieved SVR more frequently, although this difference was not statistically significant [21]. It is well known that a meta-analysis allows an unbiased pooling of available evidence regarding the efficacy of any given intervention, but two other meta-analyses [24,25] included different numbers of studies, in one [24] more than in ours, and patients with different clinical characteristics. In both meta-analyses, the researchers pooled the results from randomized trials on patients with different HCV genotypes, full papers and abstracts, thus reducing the quality and reliability of the results. The meta-analysis by Awad and colleagues’ included anti-HIV-positive patients, non-responders to previous treatment, and those treated with sub-optimal doses of Peg-IFN α-2b, and found that SVR was more frequently achieved in patients with genotype 1 or 4 treated with Peg-IFN α-2a plus ribavirin than in those receiving Peg-IFN α-2b plus ribavirin [24]. Moreover, it should be pointed out that the studies considering subgroups of patients receiving a sub-optimal or insufficient dosage of one or both drugs were not included in our meta-analysis. Also excluded were the studies that included patients with HCV infection who were non-responders to previous antiviral treatment and those with HCV/HIV coinfection, who in previous investigations showed a much lower SVR rate than therapy-naïve and anti-HIV-negative patients [2,26-28]. The meta-analysis by Zhao and colleagues pooled the results from trials on patients with genotype 1 and 4 and on those with genotype 2 and 3; they reported that SVR was more frequently associated to the use of Peg-IFN α-2a than Peg-IFN α-2b in patients with HCV genotype 2 or 3, whereas the overall effect in the subset of patients with genotype 1 or 4 was not significant [25].

A low dose of ribavirin and/or its reduction during treatment were associated to a high rate of relapse after antiviral treatment and, thus, to a low rate of SVR [29,30]. Among the trials enrolled in our meta-analysis McHutchison’s study reports the data on SVR according to the dosage and to the reduction of ribavirin during treatment, allowing a sub-analysis of the relative subgroups [19]. Considering only the patients receiving an adequate daily dose of ribavirin (≥13 mg/kg/die) in McHutchison’s trial, no difference in the achievement of SVR was found between the Peg-IFN α-2a and Peg-IFN α-2b schedules. Considering only the patients with no reduction of ribavirin during treatment, Peg-IFN α-2a compared with Peg-IFN α-2b more frequently achieved SVR, but the data of this sub-analysis are strongly impaired by the lack of information on the ribavirin dose prescribed for this subset of patients.

The present meta-analysis also evaluated the rate of adverse events leading to the discontinuation of treatment or to a reduction of the Peg-IFN dose. However, since these data for patients with HCV genotype 1 were reported only in a few trials, the rate of adverse events in the meta-analysis includes all the studies considering patients with genotype 1 or non-1. The treatment schedules were frequently burdened by adverse events that had a substantial clinical impact, with approximately 10% of patients discontinuing the treatment schedules and 6-25% receiving a reduced Peg-IFN dose because of such events. It is important to underline that the data show that Peg-IFN α-2a and Peg-IFN α-2b had similar frequencies of adverse events leading to treatment discontinuation or drug dosage reduction, but it should be noted that the adverse effects of treatment were inconsistently reported in the papers included in the meta-analysis. Although this was not a specific objective, these aspects may introduce a bias and are likely to contribute to the underestimation of the true burden of effect. This meta-analysis shows that clearly defined, standardized clinical endpoints, particularly for the adverse events, would greatly enhance the interpretation of trial evidence.

The overall quality of each trial included in the meta-analysis was particularly disappointing. In none of the studies were the observers masked to treatment and to the results, and many studies did not describe the randomization procedures in detail. Although the poor quality of the studies can affect the results and may undermine confidence in the conclusions drawn, it should be pointed out that the magnitude and direction of the results did not change in the sensitivity analysis when trials with a low quality score were excluded. Similarly, the results did not change significantly when the data from the non-randomized trials were removed. Thus, the sensitivity analysis strengthens the validity of the overall conclusion that the efficacy of Peg-IFN α-2a was similar to that of Peg-IFN α-2b. The finding that the results of the sensitivity analysis did not modify those of the meta-analysis may at least be explained in part by the fact that the McHutchison trial [19] accounted for more than two-thirds of all patients included.

The potential limitations of this meta-analysis are those of any meta-analytic venture and should be acknowledged. First, the possibility of a publication bias needs to be borne in mind, particularly in a meta-analysis based only on published studies, because “positive” studies are more likely to be submitted and published than “negative” studies, and so it is possible that other small “negative” studies have been conducted and their results never published. However, because this is a relatively new treatment, it is improbable that other “negative” studies exist. The literature search was conducted by searching multiple electronic databases, the reference lists of the retrieved manuscripts and reviews of experts in this field and was limited to articles published in the English language. We purposefully did not contact the authors of the articles included in this meta-analysis, because we wished to assess the evidence as it stands in the public domain. Finally, the need to combine results from randomized and non-randomized studies constitutes a potential limitation. However, the results of the sensitivity analysis did not show any difference in the efficacy of the treatment in relation to the type and quality between the randomized and non-randomized studies, and importantly, the absence of statistical heterogeneity or publication bias suggests that the results are robust. Despite its limitations, this meta-analysis provides the most comprehensive and updated summary of the epidemiological evidence to date on the efficacy of Peg-IFN α-2a compared to Peg-IFN α-2b, both in combination with ribavirin, in the treatment of patients with HCV genotype 1 chronic HCV.

In conclusion, this meta-analysis found a similar SVR rate between the standard schedule of Peg-IFN α-2a and Peg-IFN α-2b, both in combination with ribavirin, and therefore both treatments can be used indifferently for patients with genotype 1 chronic hepatitis C who are anti-HIV-negative and naïve to antiviral treatment.

## Abbreviations

ETR: End of Treatment Response; EVR: Early Complete Virological Response; Peg-IFN: Pegylated interferon; RVR: Rapid Virological Response; SVR: Sustained Virological Response; HCV: Hepatitis C virus; HIV: Human immunodeficiency virus; RRs: Risk ratios; SEs: Standard errors; CI: Confidence interval; AE: Adverse events.

## Competing interests

The authors declare that they have no competing interest.

## Authors’ contributions

NC was responsible for the conception and design of the study, performed the data extraction, assessed the quality, interpreted the data and wrote the manuscript. MP participated in the conception of the study, performed the data extraction and interpreted the data. CS performed the literature search and interpreted the data. GT performed the literature search and interpreted the data. ES critically revised the manuscript for important intellectual contribution. IFA was responsible for the conception and design of the study, assessed the quality of the studies, analysed the data and wrote the manuscript. All authors read and approved the final manuscript.

## Pre-publication history

The pre-publication history for this paper can be accessed here:

http://www.biomedcentral.com/1471-2334/12/357/prepub
